# Assessment of Hepatic Detoxification Activity: Proposal of an Improved Variant of the ^13^C-Methacetin Breath Test

**DOI:** 10.1371/journal.pone.0070780

**Published:** 2013-08-15

**Authors:** Hermann-Georg Holzhütter, Johan Friso Lock, Pouria Taheri, Sascha Bulik, Andrean Goede, Martin Stockmann

**Affiliations:** 1 Institute of Biochemistry, Computational Biochemistry Group, Charité - Universitätsmedizin Berlin, Berlin, Germany; 2 Department of General, Visceral- and Transplantation Surgery, Charité - Universitätsmedizin Berlin, Berlin, Germany; University of Navarra School of Medicine and Center for Applied Medical Research (CIMA), Spain

## Abstract

Breath tests based on the administration of a ^13^C-labeled drug and subsequent monitoring of ^13^CO_2_ in the breath (quantified as DOB – delta over baseline) liberated from the drug during hepatic CPY-dependent detoxification are important tools in liver function diagnostics. The capability of such breath tests to reliably indicate hepatic CYP performance is limited by the fact that ^13^CO_2_ is not exclusively exhaled but also exchanged with other compartments of the body. In order to assess this bias caused by variations of individual systemic CO_2_ kinetics we administered intravenously the test drug ^13^C-methacetin to 25 clinically liver-healthy individuals and monitored progress curves of DOB and the plasma concentration of ^13^C-methacetin. Applying compartment modelling we estimated for each individual a set of kinetic parameters characterizing the time-dependent exchange of the drug and of CO_2_ with the liver and non-hepatic body compartments. This analysis revealed that individual variations in the kinetics of CO_2_ may account for up to 30% deviation of DOB curve parameters from their mean at otherwise identical ^13^C-methacetin metabolization rates. In order to correct for this bias we introduced a novel detoxification score which ideally should be assessed from the DOB curve of a 2-step test (“2DOB”) which is initialized with the injection of a standard dose of ^13^C-labeled bicarbonate (in order to provide information on the actual CO_2_ status of the individual) followed by injection of the ^13^C-labeled test drug (the common procedure). Computer simulations suggest that the predictive power of the proposed 2DOB breath test to reliably quantity the CYP-specific hepatic detoxification activity should be significantly higher compared to the conventional breath test.

## Introduction

Non-invasive breath tests based on the stable isotope ^13^C have become a valuable diagnostic tool for several diseases. The core idea of such tests is the administration of a substrate with a labeled functional group. The substrate undergoes specific metabolization in the tissue under investigation, thus ^13^CO_2_ is released and can be quantitatively assessed in the expired breath. Breath tests are attractive for being less invasive, relatively simple and having a high patient acceptance [1], [2]. Popular examples of breath tests are the detection of Helicobacter pylori infection by ^13^C-urea breath test [3] or testing of exocrine pancreatic function by ^13^C-labeled triacylglycerides [4]. In liver surgery, breath tests are of particular relevance for the pre- and postoperative assessment of the organ function. To this end, various substrates as, for example, ^13^C-aminopyrine [5], [6], ^13^C-galactose [7], [8] or ^13^C-phenylalanine [9] can be used. Several groups [9], [10], [11], [12], [13], [14], [15], [16] prefer ^13^C-methacetin because of its non-toxicity in low doses and exclusive metabolization by the liver.

In this work we focus on the ^13^C-methacetin breath test (MBT). Hepatic (microsomal) deacylation of this drug yields as reaction products ^13^CO_2_ and the analgetic and antipyretic drug paracetamol. The diagnostic value of the conventional MBT has remained somehow limited, since oral administration of methacetin leads to a delay in kinetics by gastric emptying and absorption. Thus, the MBT is weak in discriminating between different stages of progressing fibrosis and liver cirrhosis due to larger individual differences between distinctive patients [12], [14]. However, a new intravenous test protocol with online measurement (LiMAx test) seems to enable a more precise assessment. This has been clinically validated for the prediction of postoperative outcome after liver surgery [15], [16] and transplantation [17], [18].

A general issue with all 13C-labeled breath tests is, however, that emerging ^13^CO_2_ is not directly and exclusively eliminated by respiration but partially distributed throughout the whole body. In a preliminary report [19] we found that the time curve of exhaled ^13^CO_2_ was significantly delayed compared with the kinetics of methacetin plasma washout. This finding can be reasoned by the fact that in virtually all tissues ^13^CO_2_ can be incorporated into organic molecules (^13^CO_2_-fixation) by various biochemical reactions of the intermediary metabolism. In addition, it is known that ^13^CO_2_ can be integrated in the large bicarbonate pool in blood and blood cells. Those fractions of transiently trapped ^13^CO_2_ can be liberated with some delay (e.g. ^13^CO_2_-decarboxylation) into the plasma and exhaled. Another issue is that a certain fraction of ^13^CO_2_ is not recovered in the breath because of renal clearance as H^13^CO_3_^−^ or hepatic clearance as ^13^C-labeled urea. The relative contribution of these non-respiratory clearance processes to the kinetics of ^13^CO_2_ in the blood and thus in the breath shows larger intra- and inter-individual differences [20], [21], [22] and is strongly affected by the metabolic state of the investigated patients [23].

The aim of this work was to assess the impact of individual variations in the exchange kinetics of CO_2_/bicarbonate with other body compartments on the DOB profile and to suggest a combined experimental-computational approach that enables to correct the kinetics of exhaled ^13^CO_2_ measured in the MBT for the contribution of systemic CO_2_/bicarbonate kinetics and by this to provide a more precise assessment of the actual functional capacity of the liver.

## Methods and Mathematical Modeling

### Experimental/clinical data

A group of 25 healthy volunteers were enrolled into the study after exclusion of any disease by medical history, standard laboratory and clinical examination. Descriptive characteristics see Table 1. In addition 30 patients with chronic liver diseases prior liver transplantation were included into analysis. Those patients had participated in another clinical study that has already been published elsewhere [15], [18]. Out of this group, 10 patients suffered from malignant tumors in non-cirrhotic livers, another 10 patients had biopsy proven cirrhosis but compensated function (serum bilirubin 2.7±1.7 mg/dl), finally ten patients with end-stage cirrhosis and decompensated function (serum bilirubin 4.4±3.0 mg/dl).

**Table 1:** Model parameters for individual test subjects.

parameter	unit	P1	P2	P3	P4	P5	P6	P7	P8	P9	P10	P11	P12	P13	P14	P15	P16	P17	P18	P19	P20	P21	P22	P23	P24	P25	mean	variance
k_−M_	min^−1^	4.32	3.02	4.73	4.49	1.15	3.88	1.16	2.16	3.02	4.00	3.16	1.91	3.71	1.49	3.06	1.08	1.79	0.98	3.40	2.99	5.04	0.88	1.52	3.87	4.50	**2.85**	**1.34**
k_+M_	min^−1^	0.68	0.68	0.63	0.66	0.17	0.68	0.23	0.34	1.00	0.32	0.87	0.51	0.57	0.51	0.47	0.45	0.46	0.21	1.00	0.75	0.54	0.32	0.37	0.54	0.42	**0.54**	**0.22**
k_−C_	min^−1^	0.80	0.51	0.44	0.50	0.20	0.34	0.58	0.16	0.51	0.35	0.34	0.28	0.34	0.28	0.38	0.32	0.49	0.41	0.56	0.52	0.24	0.68	0.47	0.58	0.21	**0.42**	**0.16**
k_+C_	min^−1^	0.13	0.12	0.18	0.15	0.06	0.10	0.17	0.06	0.19	0.14	0.13	0.08	0.14	0.15	0.14	0.14	0.10	0.10	0.11	0.14	0.08	0.16	0.13	0.11	0.07	**0.12**	**0.03**
k_L_	min^−1^	0.13	0.11	0.18	0.09	0.07	0.16	0.16	0.06	0.23	0.08	0.11	0.15	0.11	0.26	0.16	0.10	0.14	0.11	0.12	0.06	0.12	0.08	0.12	0.11	0.07	**0.12**	**0.05**
k_−P_	min^−1^	2.03	1.68	2.11	2.71	1.09	1.08	1.61	2.69	1.63	1.87	1.06	1.85	1.07	1.22	2.34	1.46	1.49	1.35	1.77	1.58	1.96	1.04	1.60	2.42	2.24	**1.72**	**0.50**
k_+P_	min^−1^	0.07	0.10	0.13	0.10	0.06	0.07	0.10	0.08	0.20	0.10	0.10	0.09	0.05	0.10	0.10	0.12	0.08	0.06	0.06	0.10	0.10	0.08	0.08	0.10	0.09	**0.09**	**0.03**
γ	min^−1^	0.06	0.10	0.04	0.05	0.14	0.10	0.04	0.04	0.18	0.00	0.05	0.04	0.11	0.19	0.02	0.08	0.10	0.14	0.13	0.16	0.07	0.20	0.14	0.03	0.05	**0.09**	**0.06**
k_−M_/k_+M_		6.37	4.44	7.49	6.78	6.93	5.72	4.98	6.33	3.02	12.47	3.62	3.73	6.51	2.89	6.50	2.43	3.85	4.55	3.41	4.00	9.38	2.74	4.11	7.13	10.72	**5.61**	**2.54**
k_−C_/k+C		6.21	4.41	2.45	3.27	3.30	3.27	3.49	2.73	2.70	2.55	2.56	3.54	2.47	1.79	2.75	2.33	4.71	4.27	4.85	3.66	2.86	4.36	3.72	5.11	2.84	**3.45**	**1.05**
HMC3(%)		27.34	21.90	36.57	20.50	12.35	31.98	26.38	12.03	37.99	18.26	21.03	26.00	23.60	37.16	31.74	15.34	24.01	17.93	22.77	12.17	27.04	12.39	21.62	23.59	15.41	**23.08**	**7.81**
DOB_max_	‰	22.16	25.82	43.35	27.87	29.48	41.40	32.58	28.91	41.76	30.51	33.19	39.89	35.83	56.32	41.02	31.44	28.68	28.27	24.65	21.37	42.03	21.28	29.25	24.52	29.85	**32.46**	**8.43**
DOB_maxT_	min	5.81	8.20	5.69	13.87	10.20	5.37	7.37	11.42	5.36	13.47	10.24	6.16	8.84	5.67	5.89	14.00	6.03	7.74	7.64	24.63	6.48	22.65	8.25	6.82	10.23	**9.52**	**5.03**
DOB20	min	19.88	23.69	32.47	27.14	26.23	27.63	28.12	26.77	31.34	29.55	30.58	26.81	31.62	35.21	30.55	30.56	23.28	24.35	22.53	21.14	29.39	21.20	26.30	21.99	27.26	**27.02**	**4.01**
M_0.5_	min	0.17	0.26	0.16	0.17	0.64	0.19	0.66	0.35	0.27	0.18	0.25	0.41	0.20	0.55	0.24	0.79	0.44	0.79	0.24	0.26	0.15	0.95	0.51	0.19	0.16	**0.37**	**0.24**

All healthy volunteers and patients received an intravenous injection of ^13^C-labeled methacetin (injection dose = 2 mg/kg). The amount of ^13^CO_2_ in the breath exceeding that of continuously formed endogenous ^13^CO_2_ ( = delta over baseline – DOB) was measured over 30 minutes by a modified non-dispersive isotope-selective infrared spectroscopy based device (FANci2-db16, Fischer Analyseninstrumente, Leipzig, Germany). Mean baseline ^13^CO_2_/^12^CO_2_ ratio was recorded ten minutes before injection. Six time points (1, 2, 5, 10, 20, 30 min) were selected for analysis. The two different study protocols were prior approved by the faculty's' (University Medicine Berlin - Charité; Germany) ethical review board and written informed consent was obtained from each individual.

In addition to the breath test, the healthy volunteers received analysis of blood pharmacokinetics. The concentration of ^13^C-methacetin and paracetamol in the blood was measured at six time points (2, 5, 10, 20, 30, 60 min). Blood probes were drawn in a standardized manner: Five ml were discarded and a sample of 5 ml was drawn in a serum tube. Probes were centrifuged with 1,500 g for 4 minutes and the serum aliquot was separated. Blinded probes were analyzed for ^13^C-methacetin and paracetamol by high performance liquid chromatography (HPLC). HPLC analysis was performed using a Ultrashere ODS column (250 mm×4,6 mm×5 µm; Beckman Coulter, Krefeld, Germany) with a LC-6B system (Shimadzu, Duisburg, Germany) at a flow rate of 1.5 mL/min, with UV-detection at 260 nm. Samples of 50 µl serum were mixed with 100 µl of acetonitrile/methanol solution (1∶1) and centrifuged 10,000 g for 8 minutes before HPLC. Samples of each 10 µl were applied to the analyzer. A commercial HPLC-Test-Kit for measurement of levetiracetam (Chromsystems GmbH, Munich, Germany) was used for the analysis. The Kit-conditions were modified for estimation of methacetin and paracetamol. Chromatography was performed with a LC-6B system (Shimadzu, Duisburg, Germany) at a flow rate of 1.5 mL/min, with UV-detection at 260 nm. The sensitivity was 0.5 µg/mL with proven test linearity up to a concentration of 100 µg/ml. The mean inter-assay variability was 6.8% for methacetin and 6.9% for paracetamol.

### Estimation of characteristic parameters of ^13^C-methacetin and DOB time courses

The measured time courses of plasma ^13^C-methacetin, plasma paracetamol and DOB were approximated by an exponential regression function,
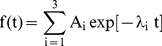
(1)with coefficients obeying the side constraints A_1_+A_2_+A_3_ = 0 for the time course of DOB and paracetamol, and A_1_+A_2_+A_3_ = M_0_ ( = initial plasma concentration of ^13^C-methacetin) for the time course of ^13^C-methacetin. From the _regression_ function we estimated the following empirical parameters: half-life of ^13^C-methacetin plasma concentration (M_0.5_), DOB peak height (DOB_max_), DOB time to peak (DOB_maxT_) and DOB at t = 20 min (DOB20).

### Compartment modeling of ^13^C-methacetin, ^13^CO_2_ and paracetamol kinetics

**Figure 1:**
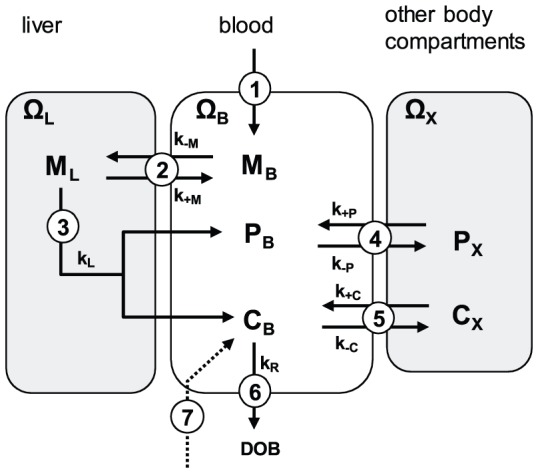
Schematic of the 3-compartment model used to describe the observed kinetics of ^13^C-methacetin (M), paracetamol (P) and ^13^CO_2_ (C) (quantified in the breath as DOB). (1) Injection of M into the blood, (2) reversible exchange of M between blood and liver, (3) hepatic metabolization of M to P and C, (4) reversible exchange of P between blood and other body compartments, (5) reversible exchange of CO_2_ between blood and other body compartments, (6) respiratory removal of M, (7) injection of H^13^CO_3_ (in the proposed novel 2DOB-method). Ω_B_, Ω_L_ and Ω_X_ denote the volume of the three compartments.

We used a 3-compartment model (Fig. 1) to describe the kinetics of ^13^C-methacetin (M), ^13^CO_2_ (C) and paracetamol (P). Note that the variable C represents the total concentration of ^13^CO_2_ and H^13^CO_3_, which are in quasi-equilibrium due to the fast carboanhydrase reaction. ^13^C-methacetin in the plasma compartment (B) is reversibly exchanged with the liver compartment (L). Exchange of methacetin with other body compartments was not considered, as its kinetic discrimination from exchange with the liver was not feasible from the measurements of plasma ^13^C-methacetin.

Hepatic formation of ^13^CO_2_ and paracetamol from ^13^C- and release into the plasma is described by a single overall reaction step. Paracetamol and ^13^CO_2_ may reversibly exchange between plasma and body compartments (X). Excretion of paracetamol was not explicitly considered in the model because the plasma half-life is 1.5–2.5 hours [24] but the potential influence of paracetamol excretion on its plasma profile can be partially captured by the parameter k_+P_. Release of plasma ^13^CO_2_ into the breath is quantified by the DOB value. Additionally, application of the proposed novel 2DOB method envisages the direct administration of H^13^CO_3_ injection into the plasma. Non-respiratory elimination of ^13^C-bicarbonate by the kidney or liver was not modeled but the potential influence of such elimination on the plasma profile and thus DOB curve can be partially covered by the parameter k_+C_.

Time-dependent changes in the compartment concentrations of ^13^C-methacetin, ^13^CO_2_ and paracetamol are governed by a system of 6 first-order differential equations:
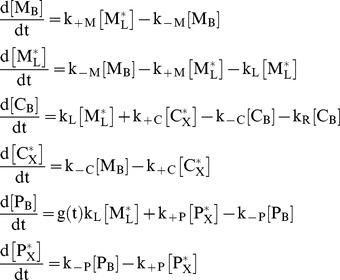
(2)The variables 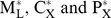 are related to the original variables of Fig. 1 M_L_, C_X_ and P_X_ through
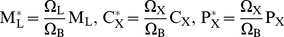
(3)where 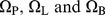 denote the volume of the respective compartment.

The function g(t) was introduced to take into account that quantifiable amounts of paracetamol occurred in the plasma with some delay compared to the rapid decline of ^13^C-methacetin:
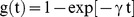
(4)Numerical estimates for the model parameters k_+M_, k_−M_, k_+C_, k_−C_, k_+P_, k_−P_ and γ obtained for the 25 subjects are given in Table 1. The estimation of parameter values was performed by combining the numerical integration of the equation system (2) by means of a 5^th^-order Runge-Kutta procedure with a non-linear regression method (Frontline Solver, Version 6.0). In these computations, the numerical value of the rate constant for respiratory CO_2_ elimination was put to 0.038 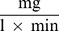 which corresponds to a total amount of 8.5 mmol CO_2_ exhaled per minute by a person having a body surface of 1.7 m^2^.

## Results

### Comparing DOB curves with plasma profiles of ^13^C-methacetin and paracetamol

First, we compared the kinetics of ^13^CO_2_ release into the breath (DOB) with the plasma profiles of ^13^C-methacetin and paracetamol. To this end, the time courses of these variables were approximated by exponential regression functions (see Methods).

**Figure 2:**
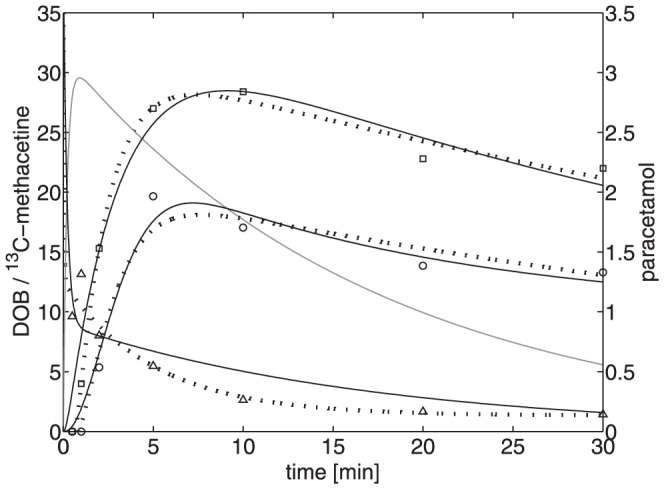
Typical time-course of plasma ^13^C-methacetin (triangles), paracetamol (circles) and DOB (squares) after intravenous injection of ^13^C-methacetin (2 mg/kg). The thin dotted lines represent best-fit curves constructed by means of an exponential regression function (1) to estimate numerical values of the characteristic parameters M_o.5_, DOB_max_, DOB_maxT_ and DOB20 (see Table 1). The solid lines represent best-fit curves obtained by fitting equation system (2) to the measured data (numerical parameter values for the shown data are for patient PX, see Table 1). The bold grey curve represents the computed time-course of the variable (Ω_L_/Ω_B_) [^13^C-methacetin] (see transformation (3)) in the liver compartment.

**Figure 3:**
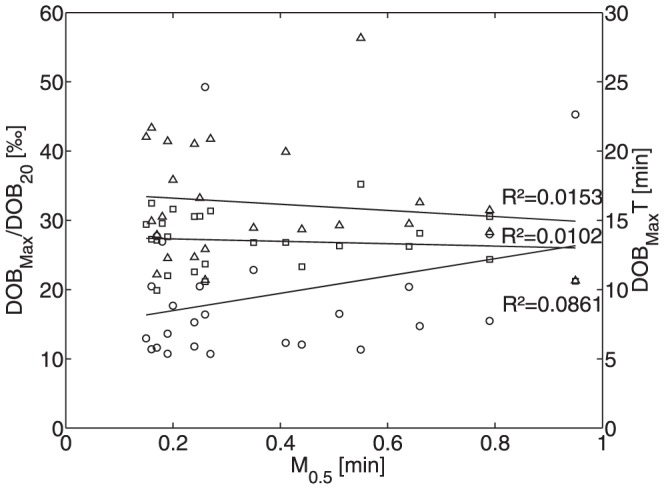
Relation between clearance of plasma ^13^C-methacetin (represented by the parameter M_0.5_ indicating the time required for a 50% drop) and the DOB curve parameter DOB_max_ (triangles), DOB_maxT_ (circles) and DOB20 (squares). Numerical values of these parameters for the 25 subjects are given in Table 1. DOB_max_ and DOB20 are given in ‰.

A typical example is shown in Fig. 2 (dotted curves). As already noted in [19], there was a significant difference between the kinetics of DOB and plasma ^13^C-methacetin. Whereas plasma ^13^C-methacetin was rapidly cleared from the plasma with a half-life of about 0.5 minutes directly after administration, elevated DOB values persisted more than 30 minutes. Moreover, for the set of 25 subjects studied the half-life of ^13^C-methacetin in the plasma, showed only a very weak correlation with DOB curve parameters (Fig. 3). These findings indicate that the early clearance rate of plasma ^13^C-methacetin merely reflects the rapid distribution of the test compound, but does not mirror its metabolization rate and thus cannot be used as reliable indicator of liver function.

### Estimation of the actual capacity for the metabolism of ^13^C-methacetin

Next, we applied the compartment model described in the methods section to estimate the actual capacity for the metabolism of ^13^C-methacetin. To this end, numerical values for all model parameters were estimated by fitting the equation system (2) to measured time-course data of plasma ^13^C-methacetin, plasma paracetamol and exhaled ^13^CO_2_ (DOB). A typical data fit is shown in Fig. 2 (solid lines). Numerical values of the model parameters for the 25 subjects are depicted in Table 1.

The bold grey curve in Fig. 2 shows (up to the linear scaling factor 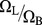, see (3)) the computed time-course of ^13^C-methacetin in the liver compartment. The peak of this concentration curve appears at a significantly earlier time point than that of the DOB curve. Such a time shift was already reported in an earlier study with ^14^C-methacetin in rats [25].

As the capacity of the liver to metabolize ^13^C-methacetin depends from both the exchange rates k_−M_ and k_+M_ and the chemical conversion rate k_L_ we decided to use as liver function parameter the fraction of administrated ^13^C-methacetin (M_0_) that within 3 minutes after its application is taken up by the liver and metabolized to paracetamol. We call this liver function parameter **F**ractional **H**epatic **M**etabolization **C**apacity (FHMC):
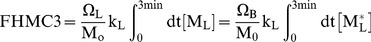
(3)With an average blood volume of 60 ml per kg body weight [26] and a ^13^C-methacetin dose of 2 mg/kg the initial plasma concentration of appearing as pre-factor in (3) was put to 
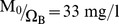
 in all calculations.

Note that the parameter FHMC defined in equation (3) overestimates the true detoxification rate of ^13^C-methacetin as our model assumes that the plasma clearance of ^13^C-methacetin is exclusively due to exchange with the liver, thereby neglecting the rapid distribution of the test drug into other compartments and tissues and its delayed release back to the plasma and subsequent metabolization in the liver.

Numerical estimates of FHMC for the 25 subjects are depicted in Table 1. On the average, about 23% of ^13^C-methacetin entering the liver within the first 3 minutes was metabolized.

**Figure 4:**
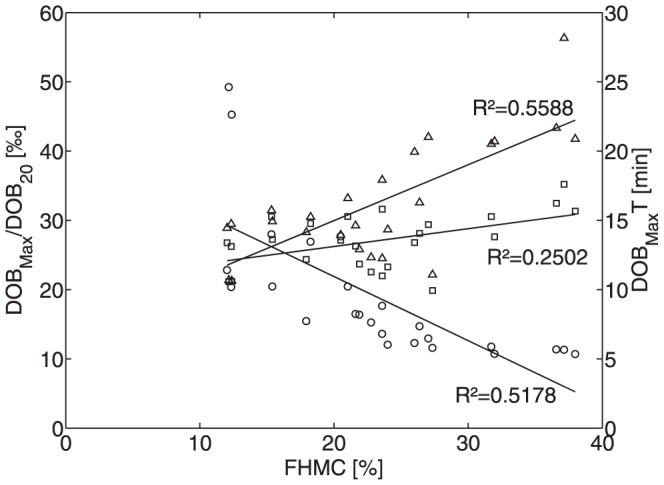
Relation between the hepatic metabolization capacity (represented by the parameter FHMC being the % share 13C-methacetin metabolized within 3 minutes) and the DOB curve parameter DOB_max_ (triangles), DOB_maxT_ (circles) and DOB20 (squares). Numerical values of these parameters for the 25 subjects are given in Table 1. DOB_max_ and DOB20 are given in ‰.

Considering that FHMC reflects the capacity of the liver to metabolize ^13^C-methacetin we asked how well this liver function parameter can be inferred from the DOB curve (see Fig. 4). The correlation of FHMC with the characteristic parameters DOB_max_ and DOB20 is indeed better than the correlation with the half-life of plasma ^13^C-methacetin (see Fig. 4). Nevertheless, relevant individual deviations from the linear regression line remain. For example, largely deviating DOB peak values of 24 and 40 may be associated with a similar FHMC value of about 22%. Hence, DOB curves as routinely monitored in the methacetin breath test may frequently provide imprecise results of the actual hepatic ^13^C-methacetin metabolization. We reasoned that the remaining discrepancies between hepatic metabolization capacity for ^13^C-methacetin (FHMC) and DOB curve parameters are due to individual variations of the exchange kinetics of ^13^CO_2_/H^13^CO_3_ between plasma and other body compartments.

### Impact of individual systemic CO_2_/bicarbonate kinetics on DOB curves

**Figure 5:**
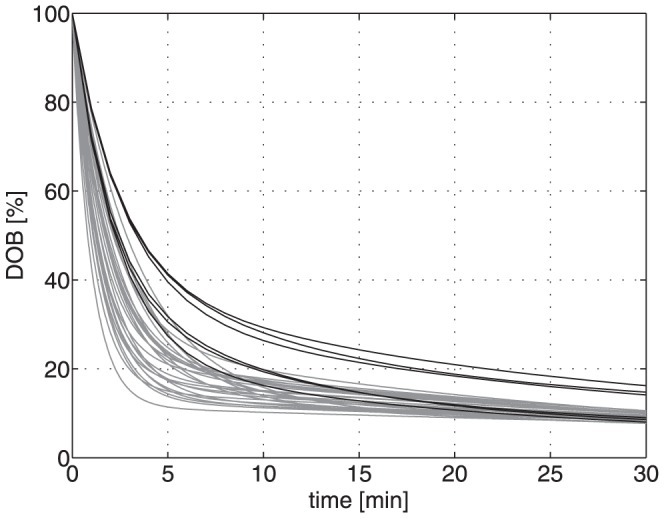
DOB_B_ curves initiated by the direct injection of 1 mmol/l 13C-bicarbonate into the plasma at time t = 0. Thin lines represent simulated DOB curves based on the kinetic model parameters k_+C_ and k_−C_ determined for the 25 subjects (see Table 1). Bold lines indicate DOB curves fitted to experimental data measured in 6 patients [20]. The DOB values are normalized to the DOB value at t = 0 (100%).

Individual variations of the exchange kinetics of ^13^CO_2_/H^13^CO_3_ between plasma and other body compartments are illustrated in Fig. 5 where we used the numerical values of the kinetic parameters k_−C_ and k_+C_ estimated for the 25 subjects to calculate DOB curves which would result from direct injection of 2.4 mg/kg bodyweight H^13^CO_3_ into the blood plasma. In the following we designate DOB curves generated by direct administration of labeled bicarbonate with DOB_B_ to distinguish them from DOB curves generated by administration of the test compound methacetin. As shown in Fig. 5, the DOB_B_ curves exhibit larger differences in the initial part between t = 0 and t = 5 minutes. At larger time points (t>15 minutes) this differences between the curves becomes smaller. All curves posses a large tail characterized by a marginal further decline. The average half-life of ^13^CO_2_ in the blood was 2.2±0.6 minutes and the average residual amount after 30 minutes was 9.2±0.9%. These values are in good agreement with values obtained from a set of 6 experimentally determined DOB_B_ curves, i.e. 2.9±0.6 minutes half-live and 11.8±3.6% residual amount, which we took from a publication on the quantification systemic CO_2_/bicarbonate kinetics in humans [20], [21]. This suggests that the numerical estimates of our model parameters k_−C_ and k_+C_ reliably reflect the ^13^CO_2_/H^13^CO_3_ exchange between plasma and other body compartments. Our simpler model for the bicarbonate washout slightly overestimates the velocity of CO_2_ exhalation compared to the more detailed models [20], [21] but yields a sufficient approximate for the short times considered in this study.

### Estimation of FHMC from conventional DOB curves of patients with different severity of liver function impairment

Conventional 60 minutes DOB curves of 30 patients prior liver transplantation were used for the validation of the developed model. As these curves do not allow the identification of neither the model parameters k_+C_ and k_−C_ (rate constants for CO2/bicarbonbate exchange) nor the model parameters k_+M_ and k_−M_ (rate constants for methacetine exchange) independent of k_L_ (CYP-dependent methacetin conversion rate) there was no other choice than neglecting individual differences in these parameters and approximating their values by the mean values obtained in the 25 liver-healthy individuals (see Table 1). Fixing the values of the parameters k_+M_ and k_−M_ to their mean is indeed not very problematic as the impact of these parameters on FHMC is largely redundant with the impact of the parameter k_L_. For example, an increase of the value of k_+M_ alone would lead to a more rapid clearance of plasma methacetine, an increase of the hepatic methacetine concentration and thus a higher value of FHMC. This effect can be almost completely nullified by a proper decrease of the value of k_L_.

**Figure 6:**
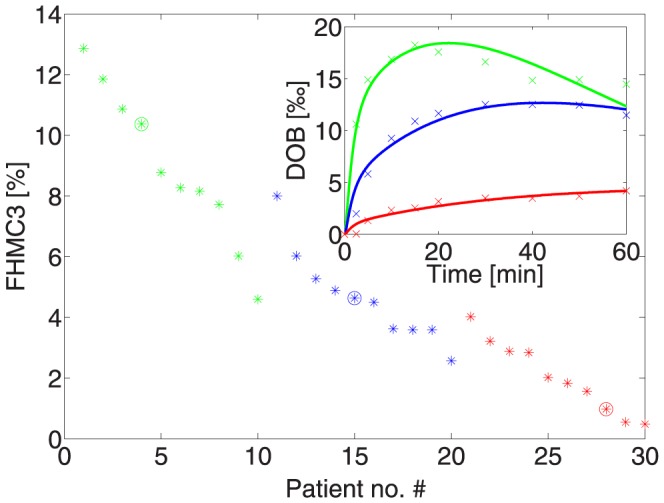
Relationship between FMHC and the severity of liver function impairment assessed by the LIMAX score FHMC values were estimated for 30 patients with different severity of liver function failure by fitting the model equation system (2) to conventional DOB curve data while fixing the values of the kinetic parameters k_+C_, k_−C_ for systemic CO_2_/bicarbonate kinetics at the mean value obtained in the 25 investigated subjects. Based on the LIMAX score the patients were arranged into three different groups of liver function failure (indicated by the colors green, blue and red) and within each group ranked with their FMHC value. The inset shows experimental and fitted DOB curves for three patients belonging to different classes of liver function impairment (indicated by a circle in the FHMC versus patient no. plot).

With this setting, the only adjustable parameters of the model are the rate constants k_L_ for the CYP-dependent methacetin conversion and the respiration rate k_R_. The resulting FHMC value is similar in all these settings. Notwithstanding the necessary simplifications used here, the compartment model provided a satisfactory description of the measured DOB curves (see inset in Fig. 6 for representative examples of such curve fits). As shown in Fig. 6, the FHMC was able to discriminate between three different classes of liver function impairment presented by the patients. This analysis demonstrates that our model can be applied to liver healthy and liver impaired individuals.

### The influence of individual variations in the hepatic detoxification kinetics on the characteristics of the DOB curve

**Figure 7:**
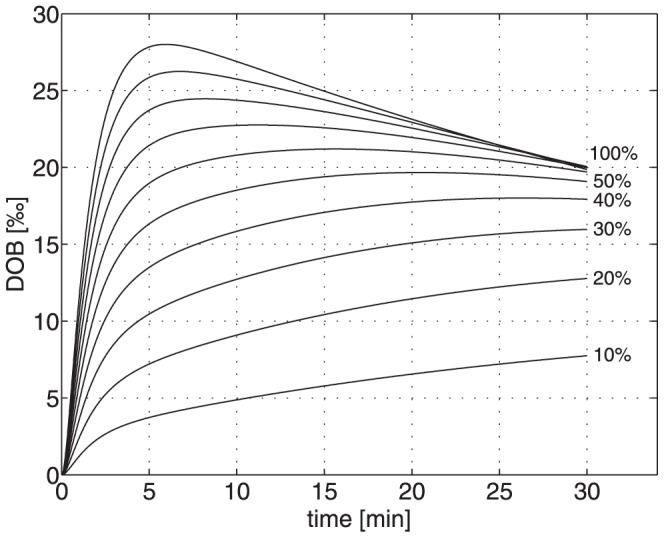
Simulated DOB (values in ‰) curves of a ‘generic’ patient at different degrees of liver impairment. The value of the metabolization rate was successively reduced in steps of 10% from the initial 100%-value (k_L_ = 0.09 min^−1^).

We assessed the consequences of the observed individual variations in the systemic CO_2_/bicarbonate kinetics on the shape of the DOB curve by computer simulations. To this end we defined a ‘generic’ liver-healthy patient by using for the parameters of the compartment model the mean values obtained across the 25 subjects (see second last column in Table 1). We simulated a gradual loss of liver function of this generic patient by reducing the numerical value of the rate constant for the conversion of methacetin to paracetamol k_L_ in 10 steps from 100% to 10% of the initial value. This resulted in the 10 different FHMC values. The associated DOB curves are shown in Fig. 7. They exhibit a successive decrease of DOB_max_, which for residual metabolization rates of less than 40% is even not defined within the observation time interval of 30 minutes, an increase of DOB_max_ and a decrease of DOB20 with decreasing metabolization rate k_L_.

**Figure 8:**
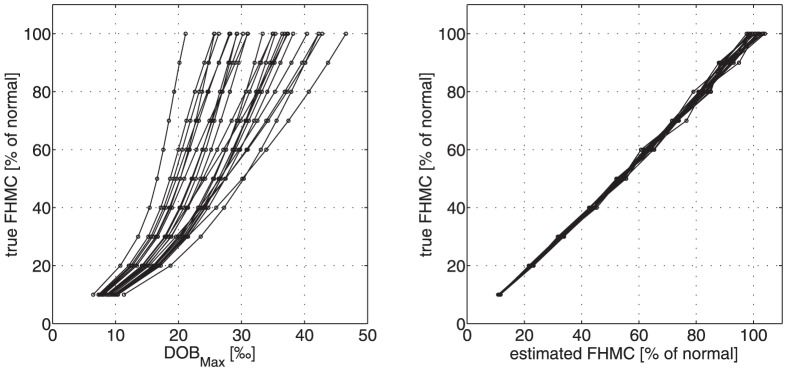
***A*** *Conventional breath test*: Relation between the true value of the liver function parameter FHMC and the characteristic parameter DOB_max_ of DOB curves. DOB curves were simulated at various degrees of liver impairment by reducing the value of the model parameter k_L_ in steps of 10%. For each degree of liver function impairment, we used the 25 different parameters pairs k_+C_, k_−C_ for systemic CO_2_/bicarbonate kinetics obtained in the 25 investigated subjects. Note that for the liver-healthy ‘generic’ patient ( = 100% metabolization capacity) the variance of DOB_max_ is 8.1 thus being 25% of the mean (36.7). ***B*** *2DOB breath test:* Relation between true and estimated values of the liver function parameter FHMC. Estimation of FHMC values was performed by fitting the compartment model to the 2-phasic DOB curve resulting from injection of ^13^C- bicarbonate followed by injection of ^13^C-methacetin (see Fig. 6).

On top, we included individual variations in systemic CO_2_/bicarbonate kinetics by using for the parameters k_−C_ and k_+C_ one after the other the numerical values obtained in the 25 subjects. This computer experiment yielded 10×25 = 250 different DOB curves for which we determined the characteristic DOB curve parameters peak height, time to peak and DOB20 from the exponential regression function (for details see legend of Fig. 8).

For each individual, a well-defined monotone relationship exists between DOB_max_ and FHCM, i.e. changes in the liver capacity should be clearly reflected by changes of DOB_max_ or related DOB curve parameters. For example, reducing the liver capacity of the patient represented by the bold curve in Fig. 8A by 50%, e.g. due to a partial hepatectomy, is predicted to lower the value of DOB_max_ by about 25% from 31 to 24. This well-defined intra-individual relation between DOB curve parameters and liver function is the prerequisite for the precise determination of individual liver function by MBT [15], [16], [17], [18].

On the other hand, inter-individual variations are large due to individual variations in the systemic CO_2_/bicarbonate kinetics largely varying values of the DOB curve parameter DOB_max_ may be associated with the same value of the liver function parameter FHMC. For example, in the worst case a numerical value of about 30 of the parameter DOB_max_ characteristic for a normal liver function can still be observed if the value of FHMC has already dropped to about 50% of the normal. Of note, the variability of DOB curve parameter values at fixed value of the liver function parameter FHMC becomes smaller with decreasing metabolization capacity of the liver. Thus, poor values of DOB parameters indeed validly reflect a poor metabolization capacity of the liver as long as the CO_2_/bicarbonate kinetics of the patient is in the range defined by the DOB_B_ curves in Fig. 5.

### 2DOB: Accounting for the Impact of individual systemic CO_2_/bicarbonate kinetics on DOB curves

**Figure 9:**
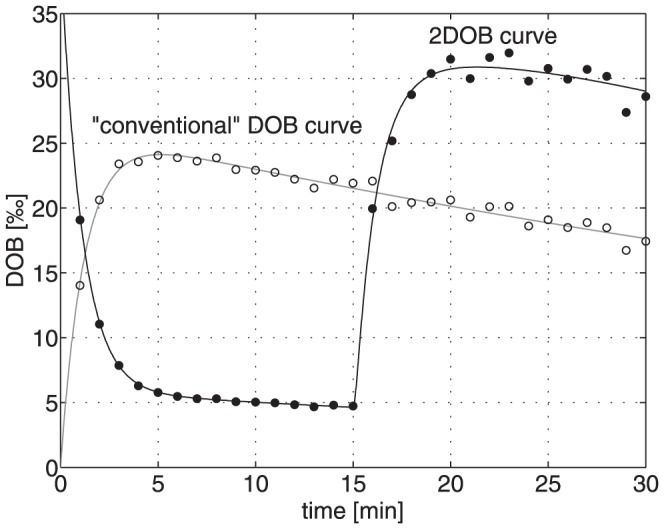
Simulated DOB (values in ‰) curves for the conventional breath test and the proposed novel 2DOB method. The conventional breath tests was simulated by generating DOB curves with the mean values of the kinetic model parameters determined in the 25 subjects (see Table 1). We imposed a random error of 15% to the simulated DOB data (open circles) and approximated these data by the exponential regression function (thin line) from which the characteristic parameters of the DOB were derived. Simulation of 2DOB breath test was done in a similar way but with the exception that first at time t = 0 a bolus of 0.1 mmol/l ^13^C-bicarbonate followed by the injection of 2 mg ^13^C-methacetin/kg bodyweight at time t = 15 min. A random error of 15% was imposed to the simulated 2-phasic DOB data (closed circles). Fitting of equation system (2) to these data yielded the model parameters k_+C_, k_−C_, k_+M_, k_−M_ and k_L_ and theoretical time-courses of DOB (bold line) and of 13C-methacetin (curve not shown) in the liver. Based on this information the numerical value of FHMC was estimated.

In order to correct the measured kinetics of exhaled ^13^CO_2_ for the impact of individual variations in the systemic CO_2_/bicarbonate metabolism allowing a more accurate assessment of the hepatic metabolization capacity we propose a novel variant of the breath test that we call 2DOB method. It envisages the administration of a defined amount of ^13^C-bicarbonate prior to the administration of the labeled test chemical. Subsequent injection of ^13^C-bicarbonate and ^13^C-methacetin results in a 2-phasic DOB curve (see bold circles in Fig. 9). The first part of this DOB curve represents the washout of ^13^C-bicarbonate from the plasma and contains information on the kinetic parameters k_+C_ and k_−C_ for the exchange of carbon dioxide between plasma and various body compartments. The second (non-monotone) part of the DOB curve starts at the time of ^13^C-methacetin administration. It represents the plasma level of ^13^CO_2_ produced by ^13^C-methacetin metabolization and on top the residual part left from the preceding ^13^C-bicarbonate administration. Therefore, the peak of the second part of the combined DOB curve is higher than the peak of the conventional DOB curve (open circles in Fig. 9). This second part of the biphasic DOB-curve contains information on the kinetic parameters k_+M_, k_−M_ and k_L_ for the exchange of ^13^C-methacetin between blood and liver and hepatic metabolization of ^13^C-methacetin. Fitting the model equations (2) to the 2-phasic DOB curves thus provides unequivocal numerical estimates for all relevant model parameters and allows the differentiation of the ^13^C-methacetin kinetics from that of systemic CO_2_/bicarbonate kinetics of the observed DOB kinetics.

To illustrate the usefulness of the proposed 2DOB approach we performed again computer simulations similar to those described in the previous section. We again generated 250 virtual patients with ^13^C-methacetin metabolization rates between 10% and 100% of the normal and 25 different parameter sets for the CO_2_/bicarbonate exchange between plasma and body compartments. However, we now initialized each computer experiment by administration of 0.1 mmol H^13^CO_3_ and monitored the resulting DOB_B_ curve over 15 minutes. Injection of ^13^C-methacetin was simulated at time t = 15 minutes and the time-course of DOB monitored again over 15 minutes (for further details of the simulation see legend of Fig. 9). Fitting of equation system (2) to the resulting 2-phasic DOB curve yielded an estimate of the liver function parameter FHMC which we compared with the actual value of FHMC used in the simulation. As shown in Fig. 8B, the 2DOB procedure yielded an almost perfect prediction of FHMC. From the *in silico* validation we conclude that the proposed 2DOB method should enable a significantly more precise estimation of the metabolization capacity of the liver and hence improve the diagnostic accuracy of breath tests.

## Discussion

^13^CO_2_-based breath tests have become an wide-spread application in the clinical diagnosis of various types of organ dysfunctions. However, one reason that up to now has prevented breath tests to enter the mainstream of clinical practice is the perception that they lack the specificity and adequate precision needed to give accurate results in real time [27], [28]. In an attempt to refine the methodology of ^13^CO_2_-based breath tests we have identified systemic distribution and elimination of CO_2_ as one crucial factor that may potentially compromise the predictive capacity of such tests. The ^13^CO_2_ released from the tissue under investigation into the blood plasma, is exchanged with numerous body compartments whereby the exchange kinetics depends on the type of the body compartment (e.g. bones exchange CO_2_ much slower than kidneys) and exhibits larger individual variations [20], [21], [22]. Therefore, we carried out a critical evaluation of the accuracy of the intravenous ^13^C-methacetin breath test to determine the functional capacity of the liver. The test results obtained during breath analysis can be influenced by variations in the individual kinetics of plasma CO_2_. As the extraction rate of the test drug from the plasma provides a more direct measure of its hepatic elimination we measured simultaneously the exhalation of ^13^CO_2_ liberated from the test drug ^13^C-methacetin and the plasma kinetics of ^13^C-methacetin and its reaction product paracetamol in 25 healthy subjects and analyzed the kinetic data by means of a pharmacokinetic compartment model.

As already reported in [19], the two time courses occur at different time scales with a rapid extraction of ^13^C-methacetin within a few minutes and a DOB profile persisting more than 60 minutes. As shown in Fig. 2, the characteristic parameters of the DOB curve commonly used as the test readouts of liver performance are not accurately correlated with the disappearance rate of ^13^C-methacetin in the plasma. According to our model-based calculations, the rapid hepatic extraction of 13C-methacetin from the plasma leads to an initial accumulation in the liver followed by a slower metabolization whereby that uptake rate of the test chemical into the liver does not allow to infer its metabolization rate. It has to be mentioned that our model does not take into account the net loss of ^13^HCO_3_^−^ from the body due to excretion by the kidney or conversion into urea in the liver. However, for the proposed short duration of the test of about 30 minutes these non-respiratory changes in the total amount of systemic ^13^HCO_3_^−^ should remain negligibly small.

We quantified the functional performance of the liver by the parameter FHMC being the percentage of the administrated dose of the test drug metabolized within the first 3 minutes. It has to be noted, that owing to the simplicity of the model the parameter FHCM represents an integral liver function parameter the value of which depends on the rate of exchange of the test compound between plasma and the hepatocyte compartment, blood perfusion of the organ, the total amount of viable and the enzymatic capacity of individual hepatocytes to detoxify the given test compound. The relative contribution of these various sources to an altered value of FHMC cannot be further quantified. Experiments with isolated hepatocytes would allow to eliminate the effect of impaired blood perfusion. Exposing cultured hepatocytes to defined perturbations such as hypoxia, oxidative stress or excessive lipid accumulation (hepatic steatosis) and measuring their capacity to detoxify ^13^C-metacetin would provide an excellent means to assess the sensitivity of FHMC against ‘mild’ alterations in the ultra-structure and biochemical functions of hepatocytes that usually precede severe liver dysfunction.

We found that DOB curve parameters, such as DOB_max_, usually used in clinical applications of the breath test to evaluate liver function showed a relatively small correlation with the liver function parameter FHMC when studied across a group of different individuals. Our analysis provided evidence that this fairly poor correlation is due to considerable individual variations in the exchange kinetics of CO_2_/bicarbonate between plasma and other body compartments.

It has to be emphasized that this finding doesn't compromise the clinical validity of the MBT as a valuable tool to identify hepatic impairment in general. As suggested by the simulation results shown in Fig. 8A, even at extreme variations in the individual short-term kinetics of systemic CO_2/_bicarbonate a reduction of the DOB_max_ to less than 50% should clearly indicate a severe impairment of the detoxifying capacity of liver. Moreover, as can be inferred from the clear monotone and quasi-linear relation between FHMC and DOB_max_ of an individual patient (see Fig. 8A) the conventional MBT is well suited for monitoring the development of liver function of individual patients [15], [16], [17], [18].

To better account for the influence of individual variations in systemic CO_2_/bicarbonate kinetics we propose a novel variant of the MBT that aims at improving the test's capability to assess the detoxifying capacity of the liver. Our approach enables to include the impact of systemic CO_2_/bicarbonate on the readout of the test, the time-course of exhaled ^13^CO_2_ (DOB). It has to be emphasized that the liver function parameter FHMC cannot be directed taken from the 2-biphasic DOB monitored in the proposed 2DOB test curve but requires the evaluation of this curve by means of the mathematical compartment model.

From the technical point of view, the proposed 2-step test requires higher effort caused by the additional injection of ^13^C-bicarbonate. On the other hand, the duration of the test could be shortened to 30 minutes (to be tested in a clinical trial). Our simulations suggest that monitoring the DOB curve over 15 minutes after injection of ^13^C-bicarbonate and another 15 minutes after injection of ^13^C-methacetin should be sufficient to gain all kinetic parameters needed for the estimation of the hepatic detoxification rate (FHMC).

As the 2DOB test required for a precise estimation of the new score FHMC has yet to be established and validated, we estimated values of FHMC from conventional DOB curves monitored in patients with different severity of liver function impairment. As these curves result from both hepatic ^13^CO_2_ formation and systemic ^13^CO_2_ distribution we had to approximate the kinetic parameters for the latter process by their mean values. Despite this necessary simplification, the FHMC enabled discrimination between different degrees of severity of liver impairment (Fig. 6) similar to existing methods. Therefore, the tailored determination of the individual FHMC based on the proposed 2DOB test variant and its normalization to basic whole-body measures like height or bodyweight (similar as in the definition of the LiMAX score) should give rise to a further significant improvement of the predictive power of the breath test.

The mathematical compartment model was chosen to balance physiological feasibility with the identification of valid model parameters. This compromise required some simplifications which, however, should be acceptable and without a significant influence on the obtained results. First, we assumed that the outflow of ^13^CO_2_ from the liver to the blood is fast enough to lump together this process and the formation inside the liver to one overall process. This assumption is supported by the finding that in isolated male rat livers perfused with erythrocyte-free solutions an equilibrium between extracellular CO_2_/bicarbonate and the liver was reached in about one minute [29]. Second, we considered only a single compartment with which plasma CO_2_/bicarbonate may exchange. Accordingly, the plasma profile of ^13^CO_2_/^13^C-bicaronate is described by a sum of two exponential functions. Earlier studies of Barstow et al. [20], [21] and Irving et al. [22] on the ^13^C-bicarbonate kinetics in humans have provided evidence that at least three different compartments with different characteristic time constants for the exchange kinetics have to be taken into account. However, over shorter time ranges of about 15–30 minutes considered in this study the error made by approximating the decline of plasma ^13^CO_2_/^13^C-bicabonate by only two exponentials remains sufficiently small. For time points larger than 30 minutes, the measured values of DOB and plasma ^13^C-methacetin showed larger deviations from the model simulations. In particular, at 60 minutes the residual level of plasma ^13^C-methacetin (being still of the order of 5% of the initial concentration) is higher than predicted by the model. We concluded that methacetin not only exchanges with the liver, but also with other body compartments. In an extended version of our compartment model including such a non-hepatic exchange of plasma methacetin we were able to describe the plasma ^13^C-methacetin profile by a sum of 3 exponentials with high accuracy. In this case, we obtained slightly lower values of the FHMC. Hence, the estimated FHMC values shown in Table 1 possibly overestimate the initial metabolization rate of the drug. Unfortunately, the extended compartment model accounting in a more detailed manner for the ^13^C-methacetin kinetics is not suited for the use in breath tests as the estimation of parameter values is not possible from the DOB curve alone but requires additionally measurements of the ^13^C-methacetin plasma profile. Third, we assumed in our simulations of the 2DOB method that ^13^C-bicarbonate and ^13^C-methaxcetione injected in a sequential manner contribute additively to the DOB value. This assumption is well justified since the endogenously produced ^13^CO_2_ is more than 10 times higher than the additionally formed ^13^CO_2_ from ^13^C-labeled bicarbonate or ^13^C-methacetin.

The validity of the proposed 2DOB approach was tested by computer simulations. The parameter values used in these simulations have been varied around physiologically meaningful values determined in 25 healthy patients. Thus, the simulation results possess a high degree of credibility. Considering the crucial role of the liver in the regulation of the acid/base-status of humans it cannot be excluded, however, that in patients with impaired liver function the individual deviations from normal CO_2_/bicarbonate exchange kinetics are larger than revealed by our analysis of liver-healthy subjects. Indeed, a clinical study with 200 cirrhotic patients showed a clear trend towards metabolic acidosis manifested by a negative base excess [30].

Finally, it has to be emphasized that the proposed method could potentially improve the diagnostic accuracy of all ^13^CO_2_-based breath tests.
